# Symptomatic hypercalcemia and lytic lesions of the skull revealing sarcoidosis: A case report

**DOI:** 10.1016/j.radcr.2024.08.115

**Published:** 2024-09-12

**Authors:** Haifa Tounsi, Wafa Skouri, Mohamed Jlidi, Sabrine Bachrouch, Haifa Mami, Yassine Kaabar, Siwar Sbaihi, Abir Chaabane, Raja Amri, Zeineb Alaya

**Affiliations:** a Department of Internal Medicine, Mohamed Tahar Maamouri University Hospital, Nabeul, Tunisia; b Department of Orthopedic Surgery, Mohamed Tahar Maamouri University Hospital, Nabeul, Tunisia; c Department of Psychiatry, Mohamed Tahar Maamouri University Hospital, Nabeul, Tunisia; d Department of Medical Biology, Mohamed Tahar Maamouri University Hospital, Nabeul, Tunisia; e Department of Medical Imaging, Mohamed Tahar Maamouri University Hospital, Nabeul, Tunisia; f Department of Anatomic Pathology, Mohamed Tahar Maamouri University Hospital, Nabeul, Tunisia

**Keywords:** Bone sarcoidosis, Skull, Osteolysis, Imaging

## Abstract

Sarcoidosis is a systemic granulomatosis of unknown etiology. Mediastinal lymph node and pulmonary involvement are the most characteristic manifestations. However, bone involvement is rare during sarcoidosis. Herein, we describe an atypical case of sarcoidosis revealed by a severe hypercalcemia and lytic lesions of the skull without pulmonary or mediastinal manifestation.

A 53-year-old woman was admitted for symptomatic hypercalcemia of 3.8 mmoL/L. The initial good course after hydration combined with a dose of intravenous zoledronate was followed by a relapse of hypercalcemia. Computed tomography (CT) scan showed 2 lytic lesions of the skull and multiple nodules and micronodules in the liver and the spleen which were of normal size. The histological examination showed a non-necrotizing granulomatous hepatitis, with no signs of malignancy. The bone marrow biopsy did not show any abnormality. Assessment for tuberculosis was negative. The diagnosis of sarcoidosis was considered. Oral prednisone therapy allowed total remission.

## Introduction

Sarcoidosis is a systemic inflammatory disease, of unknown etiology, which is characterized by a granulomatous infiltration of involved tissues. The mediastinal and pulmonary involvement is the most frequent manifestation found in 90% of cases. Extra thoracic sarcoidosis in the absence of lung involvement is uncommon. The bone sarcoidosis is rarely described in the literature. Furthermore, skull sarcoidosis is extremely rare [1].

We report an original case of sarcoidosis revealed by severe hypercalcemia with 2 lytic lesions of the skull associated to a multi-nodular liver and spleen mimicking malignancy, in the absence of thoracic manifestation. The literature dealing with skull sarcoidosis is reviewed.

## Case presentation

**Fig. 1:**
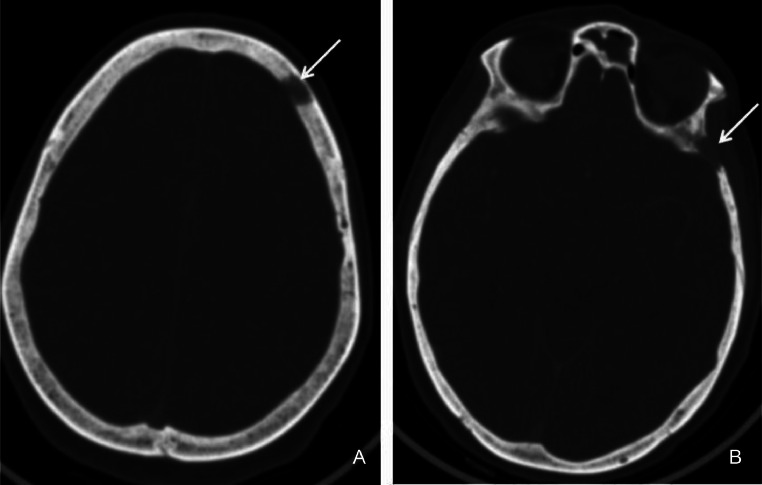
Axial section of brain CT scan showing left frontal (A) and temporal (B) osteolytic lesions.

**Fig. 2:**
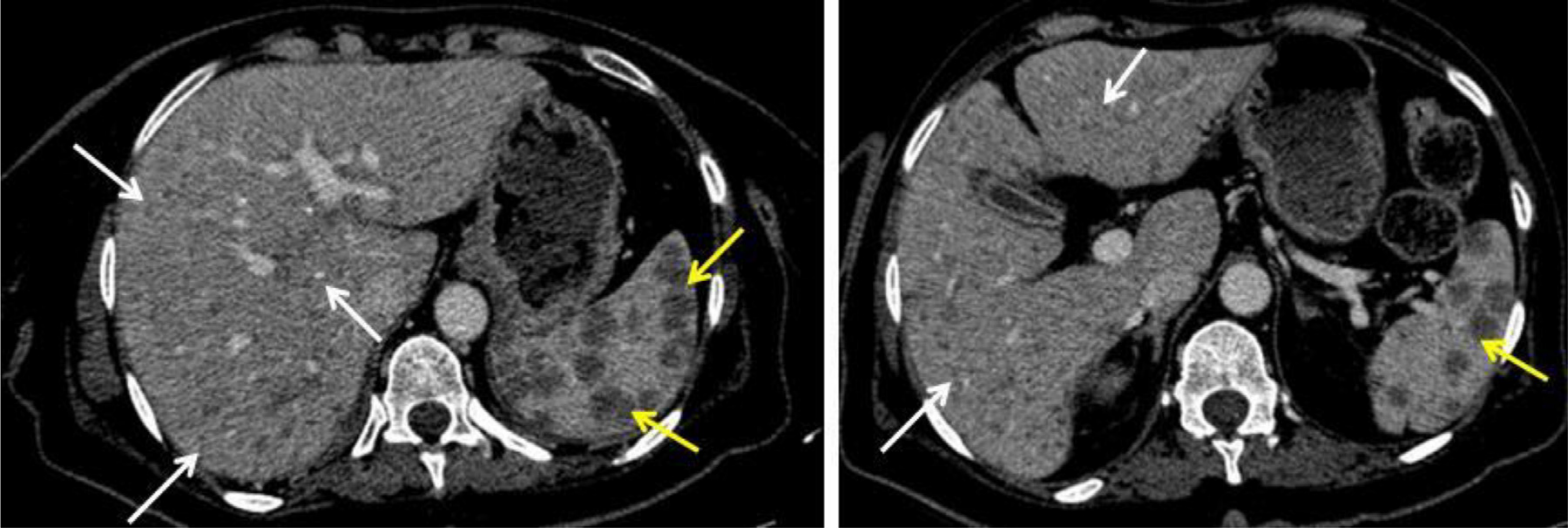
Axial section of abdominal CT scan after injection of contrast medium showing multiple hypodense nodules of the liver sparing no segment (white arrows) and the spleen (yellow arrows).

**Fig. 3:**
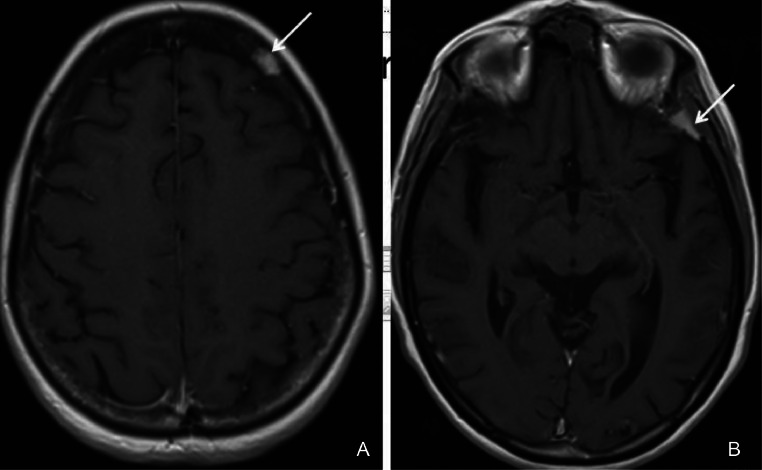
Brain MRI: Axial T1 sequence showing enhanced bone lesions after Gadolinium injection, frontal (A) and left temporal (B).

**Fig. 4:**
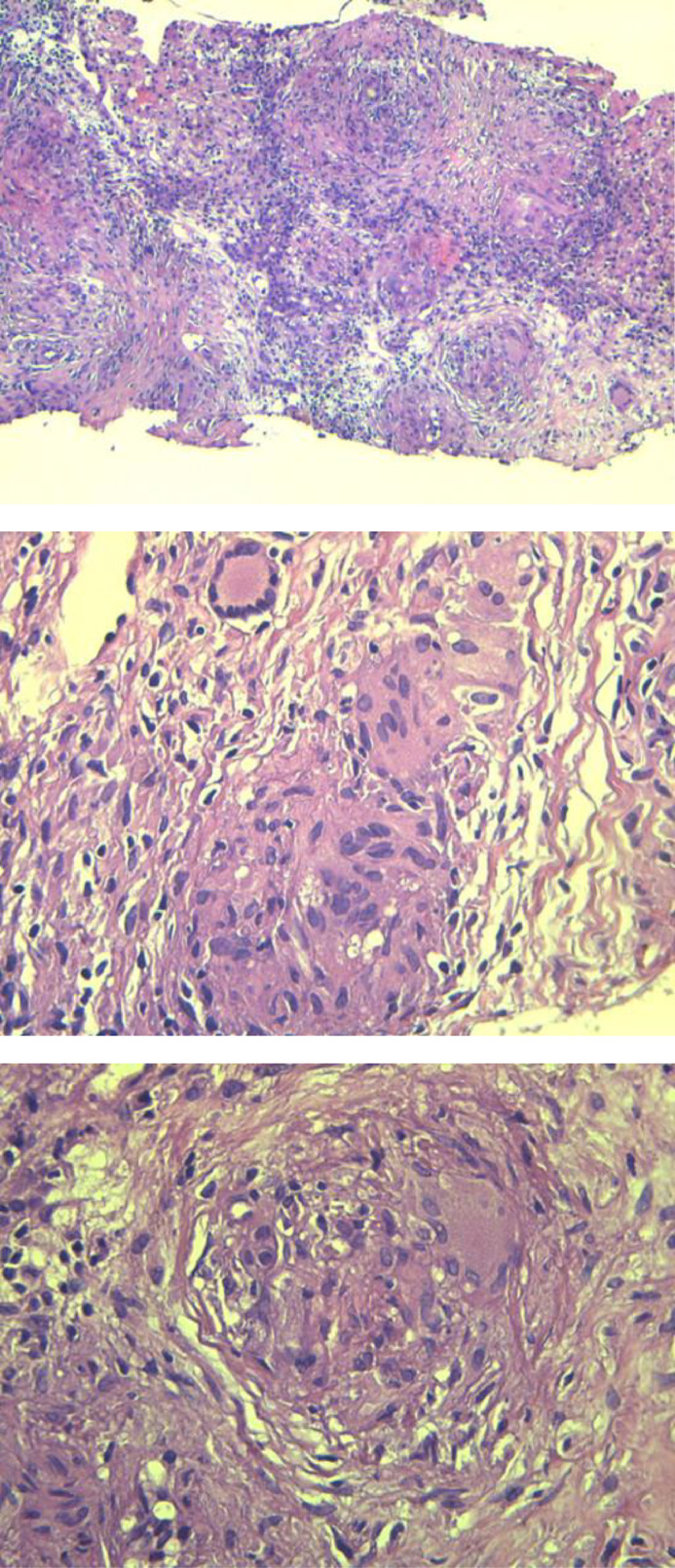
These granulomas are well-formed, characterized by a central collection of epithelioid histiocytes and multinucleated giant cells, without caseous necrosis. They are surrounded by fibrosis and lymphocytes (H&E,x200, x400).

A 53-year-old woman, with a medical history of type 2 diabetes, arterial hypertension and bipolar disorder, was admitted for asthenia, anorexia and body weight loss of 7 Kg within 4 weeks prior to admission. She also had vomiting in the last 2 days. On clinical examination, the body mass index was at 18.2 Kg/m², the patient was obnubilated. The electrocardiogram did not show any abnormality. Biologic assessment showed an elevated serum calcium level at 3.8 mmol/L with normal phosphoremia, nonregenerative normocytic normochromic anemia at 9 g/dL and acute functional renal failure with creatinine clearance at 30 mL/min. An emergent treatment with intravenous saline hydration associated with 1 dose of intravenous zoledronate was initiated. Apart from asthenia, the other clinical symptoms resolved. Serum calcium level and renal function normalized after 3 days. The parathormone was at 9 ng/L ruling out the diagnosis of hyperparathyroidism. A polyclonal hypergammaglobulinemia was found on hemoglobin electrophoresis. The 24-hour proteinuria was negative. The rest of the standard biological assessment was unremarkable. Serum and urinary protein immunoelectrophoresis did not objectify a monoclonal peak. Bence Jones' proteinuria was negative. The study of the ratio of serum light chains was normal. In imaging, the body CT scan showed 2 lytic lesions involving the left frontal and the left temporal skull bones (Fig. 1). The liver appeared inhomogeneous after contrast administration. There were multiple hepatic micronodules with hypodense appearance and sparing no segment. There were also multiples nodules in the spleen (Fig. 2). The brain magnetic resonance imaging (MRI) found the 2 lytic lesions of the skull T2-hyperintense and T1-iso-intense, enhanced after gadolinium injection with no other abnormality (Fig. 3). The dual energy X-ray absorptiometry scan showed a T-score of −2.4 at the lumber spine consisting with osteopenia. T scores at right femoral neck and the left femoral neck were respectively of −1.2 and −1.1. The mammogram was normal. The esophago-duodenal endoscopy and colonoscopy were normal. The histological and immuno-histochemical study of the bone marrow biopsy showed no abnormality. Histological examination of salivary glands was normal. A percutaneous fine needle aspiration of the liver was performed. The histopathology of the specimen was consistent with a noncaseating granulomatous hepatitis, without signs of malignancy (Fig. 4). The intradermal tuberculin test showed anergy. During this period of investigation, the patient required a total of 3 intravenous injections of zoledronate, 15 days apart, due to the recurrence of hypercalcemia despite hyperhydration and the initial normalization of the serum calcium level after the first dose of intravenous zoledronate on admission.

The diagnosis of sarcoidosis was the most likely. Oral prednisone therapy at a dose of 40 mg/day was initiated in association with oral risedronate weekly. Clinical and biological improvement were obtained with no more relapse of hypercalcemia. So, the patient was discharged. The biological control at 1 month of corticosteroid therapy showed a serum calcium at 2.32 mmoL/L and hemoglobin level at 12 g/dL. This favorable outcome comforts the diagnosis of sarcoidosis. In the subsequent 10 months follow-up of our patient, no relapse was noted. A control body CT scan is planned in 4 months.

## Discussion

Sarcoidosis is a pathology with a great polymorphism. Whether it is symptomatic or not, or acute or not, this condition can involve variable organs with a diverse clinical impact from benign to very severe [2]. The frequency of bone involvement in sarcoidosis varies depending on the author, between 1% and 36% of cases [3]. It is probably underestimated owing to asymptomatic patients who present nearly 50% of cases [2,4]. There is conflicting evidence in the literature about the preferred site of bone involvement in sarcoidosis. Indeed, bone sarcoidosis was initially described as limited to the short tubular bones of hands and feet [5,6]. Paradoxically other authors have reported a predominance of axial involvement in bone sarcoidosis, notably in the spine and the pelvis. In fact, in a monocentric study enrolling 20 patients with bone sarcoidosis, between 1994 and 2013 in Boston, the axial skeleton was involved in most cases (90%). It affected primarily the pelvis and the lumbar spine [7]. Likewise, in a French multicentric study, among the 88 patients with bone sarcoidosis, fifty-nine out of 85 patients had axial bone involvement, and 34 of them were solely affected at the axial skeletal bones. The 2 most common axial sites were the spine and the pelvis [8]. Similarly, the study of Zhou et al. [9] enrolling 64 patients with bone sarcoidosis, in the United States, found that spine was the most affected bone in 68.8% of patients, followed by pelvis (35.9%), and hands (15.6%). However, localization to the skull, like our patient, still among the rarest findings in sarcoidosis [6]. At our knowledge, there are less than 50 similar cases in the literature.

Clinically, bone sarcoidosis may manifest by a soft tissue thickening surrounding the fingers and is referred to as “sausage dactylitis”. This is commonly located in the phalanges of the hands and feet and are usually bilateral [2]. In a the French multicentric and comparative study, 46 patients among 88 with bone sarcoidosis experienced bone symptoms, mainly dactylitis (28%); and back (24%), digital (15%) and pelvic pain (9%) [8].

In routine laboratory tests, the most reported abnormalities associated to bone involvement are anemia and hypercalcemia. In the study of Zhou, anemia was more common in bone sarcoidosis group than controls (*P* = .044) and in the French multicentric study 6 out of 71 (9%) osseous sarcoidosis cases had elevated serum calcium level [8]. In our patient, hypercalcemia was severe and recurrent despite treatment with initial treatment by zoledronate. The combination of corticosteroids allowed a lasting remission. This outcome recomforts de diagnosis of sarcoidosis in our case.

At radiography, a lace-like pattern of osteolysis with thickened trabeculae and a thin cortex are characteristically seen in the small bones of the hands and feet. A periosteal reaction is typically absent [11]. Total body scintigraphy is more sensible in the assessment of bone involvement in sarcoidosis. It shows the distribution of the isotope which concentrate in the affected bones of the axial and/or in the appendicular skeleton [12]. On CT scan, the lesions had a mixed appearance with lytic, sclerotic, and permeative patterns [7]. The bone involvement in sarcoidosis can be widespread with multiple lytic lesions [6,13]. Pathologic fractures with bone collapse and misalignment may occur because of sarcoid osteolysis [11]. MRI may show a variable appearance including well-defined, focal T1-hypointense, T2-hyperintense, with varying degrees of contrast enhancement and enhancing intramedullary lesions or poorly defined infiltrative processes in the bone marrow [4,11]. MRI is more sensitive for identification of osseous abnormalities and the extent of marrow, soft-tissue, and tendon involvement. However, MRI is not reliable to distinguish osseous lesions from metastases [14]. Fluorine 18-fluorodeoxyglucose positron emission tomography (PET/CT) is also more sensitive than CT scan. Indeed, in the study of Mostard et al., PET/CT scan demonstrated bone or bone marrow involvement in more than one-third (32 of 94) of the 122 patients included. Most of the lesions (94%) could not be detected at low-dose CT [15]. These newer imaging (CT, MRI and PET/CT are very useful in the assessment of the extent of the disease and have made it possible to better objectify axial bone damage during sarcoidosis [16]. On the other hand, they also may show lung involvement, lymphadenopathies and hepatic and/or splenic nodules. Similarly to our observation, Zhou et al. noted that bone sarcoidosis was significantly associated to multi-organ involvement and higher incidence with liver, spleen, and extra thoracic lymph node involvement than controls (*P* < .05) [9].

The diagnosis of sarcoidosis may be difficult in some cases, like in our patient. It is based on 3 main criteria: a compatible presentation, the evidence of noncaseating granulomas on histological examination and the exclusion of any alternative diagnosis. Bone sarcoidosis is particularly challenging. The main differential diagnosis includes malignancies and infections, especially tuberculosis [10]. Bone biopsy is often necessary to rule out alternative diagnosis [4].

Given its rarity, there is no consensus for the treatment of bone sarcoidosis. Treatment may be not necessary in asymptomatic patients. While others with pain and bone destruction should be treated. Oral corticosteroids are the cornerstone of the treatment of bone involvement in sarcoidosis [17]. They have been proven successful in reducing both symptoms and radiological changes [4,18]. In our patient bisphosphonate alone failed to maintain serum calcium in the normal range after an initial favorable outcome. Whereas prednisone allowed a progressive and total remission. Among the 20 patients included by Sparks and al. only 45% needed treatment consisting mainly of prednisone, methotrexate and hydroxychloroquine. Only 2 patients with a refractory bone sarcoidosis needed multiple immunosuppressant including tumor necrosis factor inhibitors [7] . Zhou et al. reported that infliximab was more commonly used in bone sarcoidosis patients than controls (*P* = .009) [9].

The progression of bone lesions in sarcoidosis is unpredictable. In fact, spontaneous regression was observed with clinical and radiological involution in some cases [4,18,19]. In the case reported by Lefere, baseline MRI showed multiple large vertebral lesions that were hypointense on T1-weighted and hyperintense on T2-weighted fat-saturated sequences. On subsequent MRI examinations at yearly intervals a gradual inversion of these signal characteristics (i.e. T1-weighted hyperintensity and T2-weighted fat-saturated hypo intensity) was noted starting at the periphery of the lesions “fatty halo” This transition from oedema like to fatty like signal is a well-known favorable finding indicating healing [4]. In another recent case reported by Niederhauser, a peripheral T1-hypointense and T2-hyperintense halo appeared around a core of fatty signal. This finding coincided with a clinical disease relapse after initially successful treatment with a TNF-α inhibitor [20].

## Conclusion

In summary, bone sarcoidosis may involve any bone in the axial and/or appendicular skeleton and may be associated with multi-organs affection. Newer imaging (Scintigraphy, CT scan, MRI and PET/CT) have made it possible to better recognize bone sarcoidosis. However, it still a challenging diagnosis. Indeed, initial concern is malignancies and infections, notably tuberculosis in our country. Given its rarity, there is no consensus in bone sarcoidosis treatment. The outcome of such condition is unpredictable.

## Ethics approval

Our institution does not require ethical approval for reporting individual cases or case series.

## Patient consent

Written informed consent was obtained from the patient(s) for their anonymized information to be published in this article.
